# Geometric phase analysis of magnetic skyrmion lattices in Lorentz transmission electron microscopy images

**DOI:** 10.1038/s41598-024-62873-8

**Published:** 2024-05-29

**Authors:** Thibaud Denneulin, András Kovács, Raluca Boltje, Nikolai S. Kiselev, Rafal E. Dunin-Borkowski

**Affiliations:** 1 Ernst Ruska-Centre for Microscopy and Spectroscopy with Electrons, Forschungszentrum Jülich, 52425 Jülich, Germany; 2 Peter Grünberg Institute and Institute for Advanced Simulation, Forschungszentrum Jülich and JARA, 52425 Jülich, Germany

**Keywords:** Magnetic skyrmions, Lorentz TEM, Geometric phase analysis, Deformations, Materials science, Nanoscience and technology, Physics

## Abstract

Magnetic skyrmions are quasi-particles with a swirling spin texture that form two-dimensional lattices. Skyrmion lattices can exhibit defects in response to geometric constraints, variations of temperature or applied magnetic fields. Measuring deformations in skyrmion lattices is important to understand the interplay between the lattice structure and external influences. Geometric phase analysis (GPA) is a Fourier-based image processing method that is used to measure deformation fields in high resolution transmission electron microscopy (TEM) images of crystalline materials. Here, we show that GPA can be applied quantitatively to Lorentz TEM images of two-dimensional skyrmion lattices obtained from a chiral magnet of FeGe. First, GPA is used to map deformation fields around a 5–7 dislocation and the results are compared with the linear theory of elasticity. Second, rotation angles between skyrmion crystal grains are measured and compared with angles calculated from the density of dislocations. Third, an orientational order parameter and the corresponding correlation function are calculated to describe the evolution of the disorder as a function of applied magnetic field. The influence of sources of artifacts such as geometric distortions and large defoci are also discussed.

## Introduction

Deformation mapping is a long-standing topic in transmission electron microscopy (TEM) and various techniques have been developed based on high resolution imaging, diffraction or interferometry^1,2^. In most studies, the word *deformation* is associated with the crystal lattice formed by atoms in solids. However, statically stable topological solitons such as magnetic skyrmions^3^ can also form two-dimensional lattices in particular conditions of temperature and magnetic fields^4–6^. In chiral magnets, skyrmions are stabilized by the balance between the exchange interaction and the Dzyaloshinskii-Moriya interaction. They have raised interest because of their potential applications in the field of spintronics, for instance in racetrack memories where they are treated as information carriers^7^. Axially symmetric chiral skyrmions usually form 2D hexagonal close-packed lattices. Similarly to atomic lattices, skyrmion lattices can exhibit local deformations and crystalline defects such as dislocations and grain boundaries depending on the geometric constraints^8–10^. Defects also play a role in the disordering and the associated phase transitions that occur when increasing the external magnetic field or the temperature^11–15^. This phenomenon is often described using the Kosterlitz, Thouless, Halperin, Nelson, and Young theory (KTHNY-theory)^16–18^.

In previous studies, skyrmion lattices were investigated using real-space methods applied to Lorentz images (Fresnel defocus images), which involve detecting intensity maxima or minima, finding nearest neighbors and calculating inter-skyrmion distances^9–15^. Here, we investigate the applicability of geometric phase analysis (GPA). GPA is a widespread image analysis technique based on Fourier transforms for measuring deformation fields in TEM images^19,20^. It has been applied to conventional high resolution TEM (HRTEM) images^21,22^, scanning TEM images^23,24^, Moiré patterns^25,26^ and electron holograms^27^. In particular, it was shown that deformation maps of a dislocation in a HRTEM image calculated using GPA are in quantitative agreement with linear elastic theory^28,29^. Compared to real-space methods, GPA is straightforward computationally because there is no need to detect individual maxima, find neighbors or perform any pixel-to-pixel operation. A Fourier transform of the image is first calculated and numerical apertures are applied to a pair of Bragg spots with non-colinear reciprocal lattice *g*-vectors (a more detailed description of the method is given in Supplementary Information S1). After inverse Fourier transform, the geometric phase term $\phi _{g}(\vec {r})=-2\pi \vec {g}.\vec {u}(\vec {r})$ associated to each *g*-vector is retrieved, where $\vec {u}$ is the displacement vector^19^. The components of the displacements fields $(u_{x},u_{y})$ along orthogonal directions (*x*, *y*) are obtained by combination of the phase images. The deformation fields are then obtained using differentiation of the displacement fields

1 $$\begin{aligned} \varepsilon _{xx}&= \frac{\partial u_{x}}{\partial x}\nonumber \\ \varepsilon _{yy}&= \frac{\partial u_{y}}{\partial y}\nonumber \\ \varepsilon _{xy}&= \frac{1}{2}\left( \frac{\partial u_{x}}{\partial y}+\frac{\partial u_{y}}{\partial x}\right) \nonumber \\ \omega _{xy}&= \frac{1}{2}\left( \frac{\partial u_{y}}{\partial x}-\frac{\partial u_{x}}{\partial y}\right) \nonumber \\ \Delta _{xy}&= \frac{1}{2}\left( \varepsilon _{xx}+\varepsilon _{yy}\right) \end{aligned}$$

where $\varepsilon _{xx}$ is the horizontal deformation, $\varepsilon _{yy}$ is the vertical deformation, $\varepsilon _{xy}$ is the shear deformation, $\omega _{xy}$ is the rigid-body rotation and $\Delta _{xy}$ is the mean dilatation. In this article, deformation fields are first measured around a single dislocation and compared with linear elastic theory. Rotation fields between different crystal grains are then measured and compared with angles calculated from the density of dislocations at the grain boundaries. Finally, the transition between a well-ordered lattice and a disordered state in the presence of increasing magnetic fields is investigated using GPA.

## Results

### Displacement and deformation fields around a single dislocation

**Figure 1:**
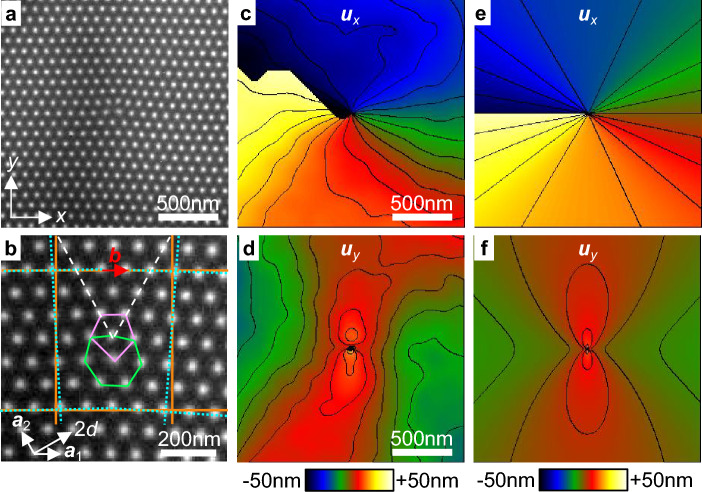
Displacement fields around a dislocation. (**a**) Fresnel image of a 5–7 dislocation in a skyrmion lattice recorded at 230 K in a lamella of FeGe with a defocus of 800 $\upmu \hbox {m}$ and in the presence of an external magnetic field of 145 mT. (**b**) Magnified image of the dislocation. (**c**,**d**) Experimental horizontal and vertical components of the displacement field ($u_{x}$,$u_{y}$) calculated from (**a**) using GPA. Contours every 5 nm between − 50 and + 50 nm. (e,f) Theoretical components of the displacement field calculated from linear elastic theory.

Figure 1a shows a Fresnel image of a skyrmion lattice in a 150 nm thick FeGe lamella recorded at 230 K with a defocus of 800 $\upmu \hbox {m}$ and in the presence of an external magnetic field of 145 mT. A basic description of the magnetic properties of the FeGe sample is given in Supplementary Information S2. Normally, each skyrmion has 6 neighbors in a hexagonal lattice. However, it is common to observe dislocations formed by pairs of 5 and 7-coordinated skyrmions^9^. Such a dislocation is present in the middle of the image and it is magnified in Fig. 1b where the heptagon and the pentagon have been drawn. The dashed lines in the top part of the image indicate the position of two half planes. The horizontal and vertical lines across the image have been traced to show displacements (dotted lines follow the lattice planes and solid lines are straight for reference). The dotted lines also form a Burgers circuit that shows the Burgers vector ${{\vec {b}}}$, which corresponds to a unit vector $\vec {a_{1}}$. The interplanar spacing *d* was estimated to be 76 nm from the position of the first-order Bragg spots in the Fourier transform (shown in Supplementary Information S1). It is essentially determined by the magnetic properties of FeGe such as the Dzyaloshinkii-Moriya and the ferromagnetic exchange interactions^30^. The magnitude of the Burgers vector was then calculated as $b=2d/\sqrt{3}=88$ nm.

Figure 1c,d shows the horizontal $u_{x}$ and vertical $u_{y}$ components of the displacement fields calculated using GPA. The spatial resolution (or the blur width) in the displacement maps is 167 nm, as determined by the 6 $\upmu \hbox {m}^{-1}$ radius of the cosine apertures used in Fourier space. The precision (or the noise) in the maps is related to the lattice fringe contrast. It was estimated to be 3 nm by calculating the standard deviation in small regions of the images after removal of linear wedges. The $u_{x}$ component varies circularly around the dislocation core. The $u_{y}$ component is positive near the middle of the image and negative on the left and right sides. This can be understood by looking at the solid and dotted lines in (b). The vertical and horizontal planes are slightly tilted anti-clockwise on the left side of the image and clockwise on the right side.

For comparison, theoretical displacement fields in Fig. 1e,f were calculated using linear elastic theory. Based on previous calculations of the magnetic energy in a skyrmion lattice, we assumed that the system can be described as elastically isotropic with a Poisson’s ratio $\nu$ of 1/3, although this value can depend slightly on the applied magnetic field^31^. In the isotropic case, the displacement fields around an edge dislocation with a Burgers vector of magnitude *b* along *x* and a dislocation line along *z* is given by^32^

2 $$\begin{aligned} u_{x}&= \frac{b}{2\pi }\left( \arctan (y,x)+\frac{xy}{2(1-\nu )(x^{2}+y^{2})}\right) \nonumber \\ u_{y}&= \frac{b}{8\pi (1-\nu )}\left( (1-2\nu )\ln (x^{2}+y^{2})+\frac{x^{2}-y^{2}}{x^{2}+y^{2}}\right) . \end{aligned}$$

The experimental (c,d) and theoretical (e,f) displacement fields are visually in good agreement, although the position of the contours is not precisely the same due to random fluctuations. Detailed comparisons are usually made using the deformation field rather than the displacement as the deformation is calculated from the derivative of the displacement and it is more sensitive to noise.

**Figure 2:**
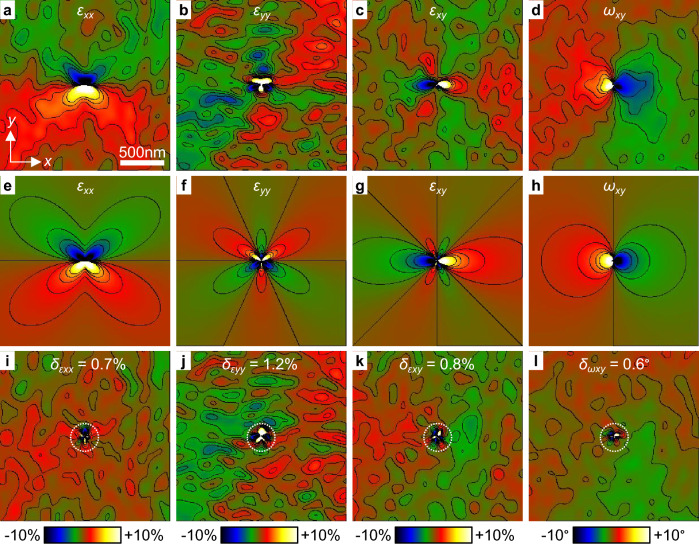
Deformation fields around a dislocation. (**a**) Horizontal deformation $\varepsilon _{xx}$, (**b**) vertical deformation $\varepsilon _{yy}$, (**c**) shear deformation $\varepsilon _{xy}$ and (**d**) rigid-body rotation $\omega _{xy}$ fields calculated from the experimental images of the displacement fields in Fig. 1c,d. Deformation contours every 1% from -5% to +5% and rotation contours every 1$^{\circ }$ from $-5^{\circ }$ to +5$^{\circ }$. (**e**–**h**) Corresponding theoretical deformation fields calculated from Fig. 1e,f. (**i**–**l**) Difference between experimental and theoretical images. The standard deviation $\delta$ is indicated on each image (the region near the dislocation core indicated by a dotted circle was excluded).

Figure 2a–d,e–h shows the experimental and theoretical deformations calculated from the displacements in Fig. 1c–f using Eq. (1). Considering the symmetry of the theoretical fields, the reference area for the calculation of the deformation was taken over the whole image. The horizontal deformation $\varepsilon _{xx}$, along the direction parallel to the Burgers vector, shows a butterfly shape with negative (compressive) deformation in the top part of the image where the extra half planes are located and positive (tensile) deformation in the bottom part. The vertical deformation $\varepsilon _{yy}$ shows a three-fold symmetry with alternating negative and positive deformations around the dislocation. The shear deformation $\varepsilon _{xy}$ and the rotation $\omega _{xy}$ show loops oriented along the horizontal direction, parallel to the Burgers vector. Visually, the shape of the experimental and theoretical deformation fields is in good agreement. In order to provide a quantitative analysis, Fig. 2i–l shows the difference between the experimental and theoretical deformation fields. The standard deviation $\delta$ was calculated and is indicated on each image. The region in the vicinity of the core was excluded from the calculation as it can show large differences^29^. These differences might be related to the reconstruction process applied to the experimental images, in particular the apertures used in Fourier space. Here, the region around the core in a radius of 150 nm, corresponding approximately to the spatial resolution of the experimental images, was excluded as indicated by dotted circles. Overall, the small values of the standard deviation $\delta \lesssim 1\%$ or $1{^\circ }$ indicate a good agreement between experiment and theory. However, besides the model of isotropic elastic theory used here, other models have been used to describe dislocations in atomic crystals including anisotropic elastic theory, Peierls-Nabarro and Foremann^33^. It would be interesting to compare these models and determine which best matches the experimental results.

### Rotation fields at grain boundaries

**Figure 3:**
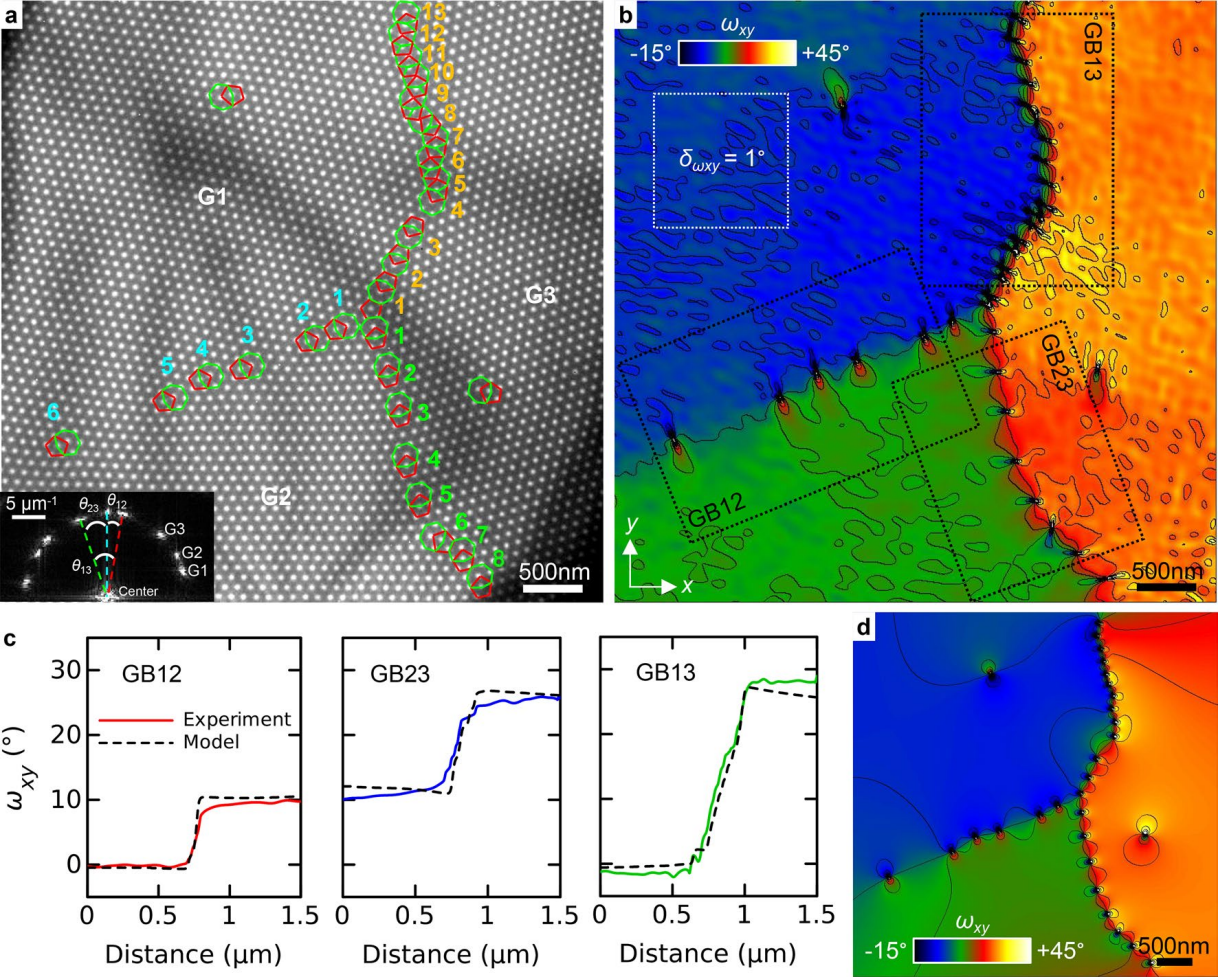
Rotation fields near grain boundaries. (**a**) Fresnel image of a skyrmion lattice showing three different crystal grains. Skyrmions with 5 (red) and 7 (green) neighbors have been drawn to indicate the position of the dislocations. The inset in the bottom left shows half of the Fourier transform of the image. (**b**) Rigid-body rotation map $\omega _{xy}$ (anti-clockwise positive) calculated using GPA with contours every 5$^{\circ }$. (**c**) Rotation profiles (solid lines) extracted from the dotted rectangles in (**b**) across the three grain boundaries. (**d**) Rotation map calculated using linear elastic theory. Corresponding profiles (dashed lines) are reported in (**c**).

Figure 3a is a Fresnel image of a skyrmion lattice obtained at 235 K in the presence of an external field of 233 mT. It shows three skyrmion crystal grains labeled G1, G2 and G3. The grain boundaries GB12, GB23 and GB13 cross at a single point near the center of the image. Grain boundaries consist of arrays of 5–7 dislocations, which are numbered on the image. This configuration was obtained by switching between the helical ground state and the skyrmion lattice state using the external magnetic field. Different grain arrangements can be spontaneously obtained during field cycling. Broad and diffuse dark lines across the image correspond to variations of the diffraction contrast related to small deformations of the FeGe lamella. These lines can be minimized locally by tilting the sample but they cannot be completely avoided over a large field of view of several micrometers. The grain boundaries GB12 and GB23 show a relatively low density with clearly separated dislocations, whereas GB13 shows a high density with strictly alternating pentagons and heptagons (in particular for dislocations 4–13). The inset shows a fast Fourier transform (FFT) of the image, which contains three different sets of spots due to the relative tilt of the grains. The angle $\theta _{13}$ in the FFT is larger than the angles $\theta _{12}$ and $\theta _{23}$, in agreement with the larger density of dislocations. Figure 3b is the rigid-body rotation map $\omega _{xy}$ obtained using GPA and using the grain G1 as the reference. Sufficiently large cosine masks of 6.5 $\upmu \hbox {m}^{-1}$ radius including the three spots have been used in Fourier space to map the three grains. Figure 3c shows three rotation profiles extracted from the maps perpendicular to the grain boundaries and averaged along the grain boundaries as shown by dotted rectangles in Fig. 3b. Rotations of $\omega _{xy}=10{^\circ }$, $15{^\circ }$ and $30{^\circ }$ were measured for GB12, GB23 and GB13 respectively, with a precision of $\delta _{\omega xy}=\pm 1{^\circ }$ given by the standard deviation of the rotation in a uniform region (white dotted square in G1).

In the case of a symmetric and low-angle ($\lesssim 15{^{\circ }}$) grain boundary, the tilt $\theta$ between two grains is related to the distance *D* between the dislocations following^34^

3 $$\begin{aligned} \theta =2\arcsin \left( \frac{b}{2D}\right) \,. \end{aligned}$$

By counting the number of dislocations and measuring the length of the low-angle GB12 and GB23, we found mean distances of $D=485$ nm and 320 nm respectively, which correspond to tilt angles of $\theta =10.4{^\circ }$ and 15.8$^{\circ }$. These values are in good agreement with the GPA measurements. However, the grain boundaries are not perfectly straight and the distance between the dislocations and their orientation are not constant. To provide a more accurate model, we have calculated a rotation map in Fig 3d by summing the rotation fields of individual dislocations. In isotropic elastic theory, the rotation field of an edge dislocation can be described in polar coordinates ($r,\varphi$) with respect to $\vec {b}$ as^35^

4 $$\begin{aligned} \omega =-\frac{b\cos (\varphi )}{2\pi r}\,. \end{aligned}$$

To create the model, the position and the orientation of the dislocations were measured from the experimental images. Profiles extracted from the model across the grain boundaries (dashed lines) have been reported in Fig. 3c. It can be observed that there is a general good quantitative agreement between the experiment and the model. Some differences can however be observed, for instance along GB13 in the region close to the triple junction (dislocations 1 to 4), the experimental rotation field shows a larger density of loops than the modeled one. Further studies could be carried to understand how the grain boundaries and their rotation fields evolve as a function of temperature, as in Ref. ^9^. Dislocations and grains can become more mobile closer to the Curie temperature due to thermal agitation.

### Evolution of deformation fields in magnetic field series

**Figure 4:**
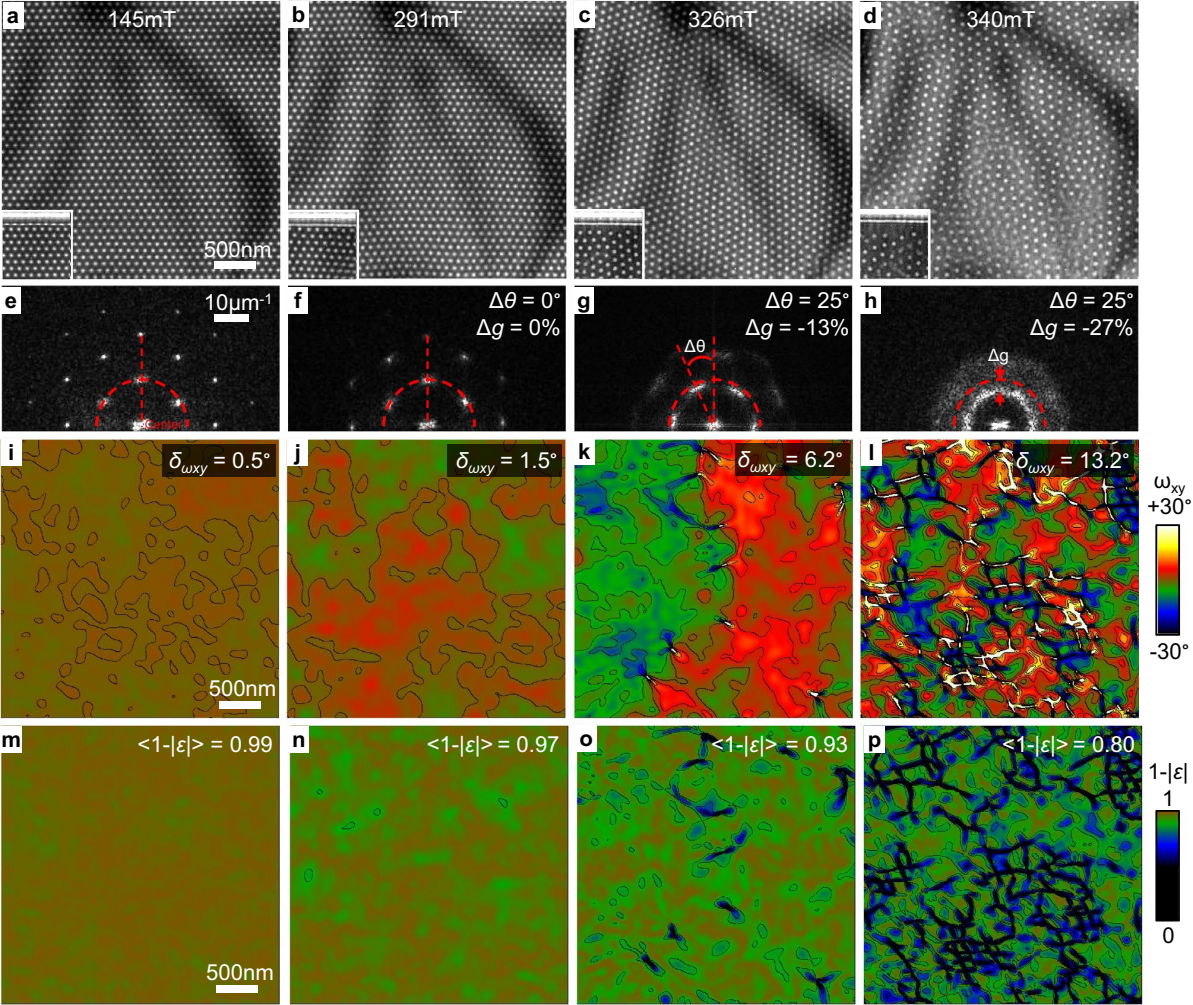
Local orientational order mapping in applied magnetic field series. (**a**–**d**) Fresnel images obtained at 230 K in the presence of external magnetic fields of 145 mT, 291 mT, 326 mT and 340 mT. The insets in the bottom left corner show an image at an edge of the sample. (**e**–**h**) Corresponding Fourier transforms. (**i**–**l**) $\omega _{xy}$ rigid-body rotation maps with contours every 5$^{\circ }$ and (**m**–**p**) $1-|\varepsilon |$ *hexagonality* maps with contours every 0.05 between 0.8 and 1. Maps were calculated using cosine apertures of 6 $\upmu \hbox {m}^{-1}$ radius in Fourier space. The standard deviation $\delta _{wxy}$ and the average value $<1-|\varepsilon |>$ are indicated on each map.

Figure 4a–d shows Fresnel images of a skyrmion lattice obtained at 145 mT, 291 mT, 326 mT and 340 mT. The insets show images of the skyrmion lattice at the edge of the sample. The images have been manually aligned and corrected for the change of magnification and rotation due to the different excitations of the objective lens. The increase of the field induces a transition from a well-ordered state (*crystalline* or *solid* phase) to a disordered state (*amorphous* or *liquid* phase). Figure 4e–h shows the corresponding Fourier transforms of (a–d) where the variations in angle $\Delta \theta$ and amplitude $\Delta g$ of a *g*-vector with respect to the first FFT are indicated. At low fields (145 mT and 291 mT), the spots in the Fourier transform are sharp and the lattice is aligned with respect to the edge of the sample (see insets in Fig. 4a,b). When increasing the field (326 mT and 340 mT), the Bragg spots in the FFT become more diffuse due to the formation of defects and domains. The orientation of the lattice appears decorrelated from the edge and an average rotation of $\Delta \theta =25{^\circ }$ is measured. In addition, the average distance between the skyrmions increases, as evidenced by the decrease of amplitude of the *g*-vector with $\Delta g=-13\%$ at 326 mT and $-27\%$ at 340 mT.

The evolution of the disorder in a hexagonal skyrmion lattice as a function of the external magnetic field can be described using the local orientational order parameter

5 $$\begin{aligned} \Psi _{6}(r_{i})=\frac{1}{N_{i}}\sum _{j=1}^{N_{i}}\exp (i6\theta _{ij}) \end{aligned}$$

where $r_{i}$ is the position of a skyrmion, $N_{i}$ is the number of nearest neighbors and $\theta _{ij}$ is the angle between the line connecting the central skyrmion *i* and its neighbors *j* with respect to an arbitrary axis^36^. The modulus of $\Psi _{6}$ represents the degree of *hexagonality* with $|\Psi _{6}|=1$ for a perfect hexagonal structure and $|\Psi _{6}|<1$ when it deviates from the hexagonal symmetry. The argument of $\Psi _{6}$ divided by 6 represents the local orientation or the *mosaicity*. Maps of $|\Psi _{6}|$ and $\arg (\Psi _{6})/6$ obtained conventionally in real space are shown in Supplementary Information S3 for reference. Here, similar maps were obtained using GPA. Orientation maps were obtained using the rigid-body rotation $\omega _{xy}$ as in the previous section. *Hexagonality* maps were obtained by summing the different deformation components, assuming that any deformation can reduce the hexagonal symmetry, except in the case of a pure dilatation $(\varepsilon _{xx}+\varepsilon _{yy})/2$. Therefore, horizontal $\varepsilon _{xx}$ and vertical $\varepsilon _{yy}$ deformations were first subtracted $(\varepsilon _{xx}-\varepsilon _{yy})/2$ to exclude dilatations. By adding the shear, we obtain a total deformation $|\varepsilon |=|(\varepsilon _{xx}-\varepsilon _{yy})/2|+|\varepsilon _{xy}|$ and the parameter $1-|\varepsilon |$ that takes a value of 1 for a perfect lattice with no deformation and less than 1 in the presence of deformations.

Figure 4i–l,m–p shows the $\omega _{xy}$ and $1-|\varepsilon |$ maps for the different magnetic fields. The standard deviation of the rotation $\delta _{wxy}$ and the mean value of the *hexagonality* parameter $<1-|\varepsilon |>$ are indicated on the maps. At low fields (145 mT and 291 mT), the maps show essentially some random fluctuations. $\delta _{wxy}$ is small (respectively 0.5$^{\circ }$ and 1.5$^{\circ }$) and $<1-|\varepsilon |>$ is close to 1 (0.99 and 0.97). At 326 mT, the rotation map shows two large domains on the left and right sides of the image with a relative tilt of approximately 10$^{\circ }$. The *hexagonality *map shows the position of the dislocations where the values are significantly lower than 1 along the grain boundary. At 340 mT, multiple small domains with different orientations are visible with a large density of dislocations. Consequently, $\delta _{wxy}$ increases (6.2$^{\circ }$ at 326 mT and 13.2$^{\circ }$ at 340 mT) and $<1-|\varepsilon |>$ decreases significantly (respectively 0.93 and 0.80).

**Figure 5:**
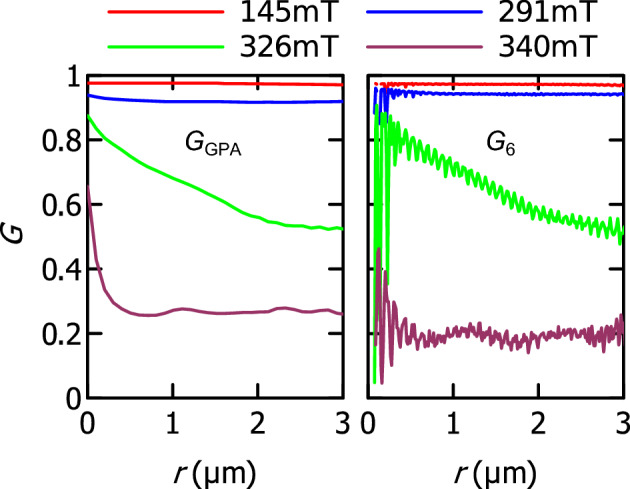
Orientational correlation functions. Plots of the orientational correlation functions $G_{\textrm{GPA}}$ and $G_{6}$ as a function of distance for different applied magnetic fields. Corresponding $\Psi _{\textrm{GPA}}$ and $\Psi _{6}$ data are shown in Fig. 4 and Supplementary Information S3.

The investigation of long-range or short-range order and the identification of different phases such as *solid*, *hexatic* and *liquid*, can be carried out based on the decay of the orientational correlation function

6 $$\begin{aligned} G_{6}(r)=\frac{1}{N_{r}}\sum _{i,j}^{N_{r}}\Psi _{6}(r_{i})\Psi _{6}^{*}(r_{j}) \end{aligned}$$

which sums over the $N_{r}$ skyrmion pairs separated by a distance *r*^36^. Here, complex images $\Psi _{\textrm{GPA}}(r)=(1-|\varepsilon |(r))\exp (i6\omega _{xy}(r))$ were first created from the GPA maps to calculate the corresponding correlation function $G_{\textrm{GPA}}(r)=1/N_{r}^{\textrm{pix}}\sum _{i,j}^{N_{r}^{\textrm{pix}}}\Psi _{\textrm{GPA}}(r_{i})\Psi _{\textrm{GPA}}^{*}(r_{j})$ where $N_{r}^{\textrm{pix}}$ is the number of pixel pairs separated by a distance *r*. Figure 5 shows the plots of $G_{\textrm{GPA}}$ and $G_{6}$ corresponding to the images in Fig. 4 and Supplementary Information S3. Overall, the profiles in the two plots show similar trends and are in good quantitative agreement. High frequency oscillations in the $G_{6}$ plots are due to the fact that the function is calculated over skyrmion pairs whereas $G_{\textrm{GPA}}$ is calculated over pixel pairs. At 145 mT and 291 mT, both plots show constant profiles close to 1 indicating a long-range orientational order, which is characteristic of a *solid* phase. At 326 mT, the profiles decay continuously from 0.9 to 0.6 at 3 $\upmu \hbox {m}$ distance. It suggests that a degree of organization persists over intermediate distances, which points towards a quasi-long range order and an intermediate phase (possibly* hexatic*). At 340 mT, the profiles decay rapidly to low values of 0.2-0.3 at 0.5 $\upmu \hbox {m}$ distance and then remain stable. It points towards a short-range order characteristic of a *liquid* phase. However, the fact that the function shows a plateau for large distances can indicate that some *solid* pockets remain inside the *liquid* phase.

## Discussion

GPA was used to measure deformation and rotation fields around defects in Fresnel images of skyrmion lattices. The results were in good agreement with theoretical models and values obtained using other methods. There are however some possible problems and artifacts that can be discussed. First, as is common for magnetic imaging, diffraction contrast should be minimized to avoid changing the magnetic contrast, for instance by tilting away from a major crystallographic axis. Second, non-linear distortions related to the projection system can introduce errors and should be analyzed in a non-deformed area^37^. Here fortunately, the analysis of a perfect skyrmion lattice showed no significant changes of magnification and rotation across the field-of-view (see Supplementary Information S4). Third, the defocus should be chosen carefully and large defoci should be avoided because of delocalization effects. Simulated Fresnel images calculated from a micromagnetic model (see Supplementary Information S5) have shown that deformation fields calculated from a Fresnel image with a defocus larger than 1.5 mm can differ from deformation fields calculated from the magnetization. In future studies, GPA could be applied to in-focus magnetic phase images obtained using off-axis electron holography for instance. In this way, artifacts related to the defocus could be avoided and direct comparisons between deformation fields, magnetic induction fields and possibly magnetization fields could be made^38^.

Some physical aspects of skyrmion lattices can also be discussed. For instance, if the chirality of the skyrmion lattice changes in some regions of the image as in Ref. ^8^, the contrast of the skyrmions will be inverted. This will most likely induce artefacts in the reconstructed deformation maps at the grain boundary. Skyrmions are also flexible objects and can show strongly deformed shapes at a dislocation core^39^. The influence of the skyrmion shape on the deformation field around a dislocation could be investigated in more details. We also hope that this method will be useful to study skyrmion lattices in samples of different geometries as in Ref. ^40,41^ for example. As the skyrmion lattice tend to align with the edges of the sample, geometric constraints could lead to different arrangements with different deformation fields. In addition, in thin samples, skyrmions can have a complicated three-dimensional magnetic texture that is not purely Bloch-type but shows also Néel-type components near the surfaces, so-called surface twist effect^42–44^. Systematic studies as a function of the thickness of the lamella could be carried out to understand the influence of the surfaces on the skyrmion lattice. However, this can require a careful interpretation of the images as both the magnetic textures and the Fresnel contrast can depend on the thickness of the sample. Atomic crystalline defects created during crystal growth or by ion implantation can also influence magnetic textures. GPA could be applied to both Lorentz TEM and high resolution TEM images obtained on the same sample to find correlations between the skyrmion lattice and the atomic lattice.

## Conclusion

We have studied deformations in skyrmion lattices in a sample of FeGe by applying GPA to Lorentz TEM images. Deformation fields were measured around a single dislocation and were found to be in good agreement with deformation fields calculated using linear elasticity. Rotations were measured at the boundaries between skyrmion crystal grains and were also in accordance with a numerical model taking into account the distribution of the dislocations. Finally, an orientational order parameter and the corresponding correlation function were obtained from GPA maps. They were used to evaluate the disorder in a skyrmion lattice as a function of the applied magnetic field and study the different phases.

## Methods

Experiments were carried out using a Thermo Fisher Scientific Titan TEM equipped with a Schottky field emission gun operated at 300 kV, a CEOS image aberration corrector and a 4k$\times$4k Gatan K2-IS direct electron detector^45^. The microscope was operated in Lorentz mode by using the first transfer lens of the aberration corrector as the main imaging lens. The objective lens was used to apply magnetic fields perpendicular to the sample which were precalibrated using a Hall probe. A liquid-nitrogen-cooled specimen holder (Gatan model 636) was used to vary the sample temperature. The Digital Micrograph software (DM Gatan) and a freely available GPA plugin for DM were used to calculate displacement and deformation maps^46^. A TEM lamella of FeGe^38,47^ was prepared from a bulk crystal using 30 kV focused Ga$^{+}$ ion beam sputtering in a scanning electron microscope (FIB-SEM) FEI Helios dual-beam platform. The last thinning step was carried out at 5 kV and the thickness of the lamella was estimated to be 150 nm.

## Supplementary Information

Supplementary Information.

## Data Availability

The data that support the findings of this study are available from the corresponding author upon reasonable request.
