# Supplementary material for: Geometric phase analysis of magnetic skyrmion lattices in Lorentz transmission electron microscopy images
Source: Sci Rep. 2024 May 29;14:12286. doi: 10.1038/s41598-024-62873-8 (PMC11637029; doi:10.1038/s41598-024-62873-8)
Supplement: Supplementary file 1 — Supplementary Information. [file 41598_2024_62873_MOESM1_ESM.pdf]

Geometric phase analysis of magnetic skyrmion lattices in Lorentz transmission electron microscopy images

Supplementary Information

Thibaud Denneulin[1], András Kovács[1], Raluca Boltje[1], Nikolai S. Kiselev[2] and Rafal E. Dunin-Borkowski[1]

[1] *Ernst Ruska-Centre for Microscopy and Spectroscopy with Electrons, Forschungszentrum Jülich, 52425 Jülich, Germany.*

[2] *Peter Grünberg Institute and Institute for Advanced Simulation, Forschungszentrum Jülich and JARA, 52425 Jülich, Germany.*

Supplementary Information 1: Basics of geometric phase analysis

The intensity $I(\vec{r})$ at a position $\vec{r}$ in an image composed of different sets of lattice fringes can be described as a sum of sinusoidal functions

$$I(\vec{r}) = \sum_g A_g(\vec{r}) \cos(2\pi\vec{g}.\vec{r} + \phi_g(\vec{r})) \,, \tag{S1}$$

where $\vec{g}$ is the reciprocal lattice vector of a set of fringes of amplitude A_g and phase ϕ_g [1]. A Fourier transform is performed and an aperture is placed around a Bragg spot $\vec{g}$. The spot is then shifted to the center of Fourier space and an inverse Fourier transform is performed, which leads to a complex image $C(\vec{r})$ of expression

$$C(\vec{r}) = A_g(\vec{r}) \exp(i\phi_g(\vec{r})) \,. \tag{S2}$$

The amplitude and the phase terms can then be separated following

$$\begin{aligned} A_g(\vec{r}) &= \sqrt{Im(C(\vec{r}))^2 + Re(C(\vec{r}))^2} \,, \\ \phi_g(\vec{r}) &= \arctan\left(\frac{Im(C(\vec{r}))}{Re(C(\vec{r}))}\right) \,, \end{aligned} \tag{S3}$$

where $Im(C(\vec{r}))$ and $Re(C(\vec{r}))$ are the imaginary and real components. After reconstruction of the phase image, a reference area can be chosen where the phase wedge is set to zero. Assuming that the lattice fringes are displaced locally by a small amount $\vec{u}(\vec{r})$, the *geometric phase* is defined [2]

$$\phi_g(\vec{r}) = -2\pi\vec{g}.\vec{u}(\vec{r}) \,. \tag{S4}$$

In order to obtain the two components of the displacement vector $\vec{u} = (u_x, u_y)$ along two orthogonal directions (x, y) in the image, two distinct sets of lattice fringes with non-colinear vectors $\vec{g_1} = (g_{1x}, g_{1y})$ and $\vec{g_2} = (g_{2x}, g_{2y})$ are reconstructed. The associated phases ϕ_{g1} and ϕ_{g2} are given in a matrix form

$$\begin{pmatrix} \phi_{g1} \\ \phi_{g2} \end{pmatrix} = -2\pi \begin{pmatrix} g_{1x} & g_{1y} \\ g_{2x} & g_{2y} \end{pmatrix} \begin{pmatrix} u_x \\ u_y \end{pmatrix} \,. \tag{S5}$$

The displacements are then

$$\begin{pmatrix} u_x \\ u_y \end{pmatrix} = -\frac{1}{2\pi} \begin{pmatrix} g_{1x} & g_{1y} \\ g_{2x} & g_{2y} \end{pmatrix}^{-1} \begin{pmatrix} \phi_{g1} \\ \phi_{g2} \end{pmatrix} \tag{S6}$$

with

$$\begin{pmatrix} g_{1x} & g_{1y} \\ g_{2x} & g_{2y} \end{pmatrix}^{-1} = \frac{1}{g_{1x}g_{2y} - g_{1y}g_{2x}} \begin{pmatrix} g_{2y} & -g_{1y} \\ -g_{2x} & g_{1x} \end{pmatrix} \,. \tag{S7}$$

The horizontal deformation ε_{xx}, vertical deformation ε_{yy}, shear deformation ε_{xy}, rigid-body rotation ω_{xy} fields and mean dilatation Δ_{xy} are then obtained by numerical differentiation of the displacement fields following

$$
\begin{aligned}
\varepsilon_{xx} &= \frac{\partial u_x}{\partial x} , \\
\varepsilon_{yy} &= \frac{\partial u_y}{\partial y} , \\
\varepsilon_{xy} &= \frac{1}{2}\left(\frac{\partial u_x}{\partial y} + \frac{\partial u_y}{\partial x}\right) , \\
\omega_{xy} &= \frac{1}{2}\left(\frac{\partial u_y}{\partial x} - \frac{\partial u_x}{\partial y}\right) , \\
\Delta_{xy} &= \frac{1}{2}(\varepsilon_{xx} + \varepsilon_{yy}) .
\end{aligned}
\tag{S8}
$$

In this article, deformations are defined in percent and rotations in degrees. Anticlockwise rotations are defined as positive and clockwise rotations as negative.

Figure S1(a) shows an example of a Fresnel image that contains a dislocation (same image as in Fig. 1(a) of the main article). The Fourier transform is shown in Fig. S1(b). Cosine apertures of 6 μm^{-1} radius were applied to two non-colinear Bragg spots with reciprocal lattice vectors $\vec{g_1}$ and $\vec{g_2}$ as shown schematically by red circles. Figure S1(c,d) shows the sets of lattice fringes obtained after inverse FFT and Fig. S1(e,f) shows the corresponding geometric phase images ϕ_{g1} and ϕ_{g2}. The phase varies monotonically in the circular direction around the dislocation core. The displacement and deformation fields calculated from these phase images are shown in Fig. 1 and 2 of the main article.

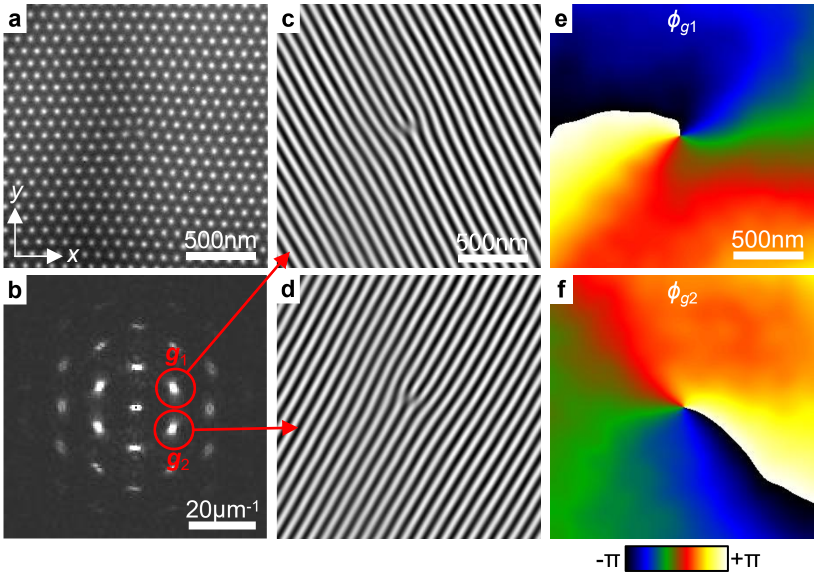

Figure S1: **Reconstruction of geometric phase images.** (a) Fresnel image of a dislocation in a skyrmion lattice recorded at 230 K with a defocus of 800 μm and in the presence of an external magnetic field of 145 mT. (b) Fourier transform of (a). Numerical apertures indicated by red circles have been placed onto two Bragg spots with vectors $\vec{g_1}$ and $\vec{g_2}$. (c,d) Images of the two sets of lattice planes obtained after inverse Fourier transform. (e,f) Reconstructed geometric phase images ϕ_{g1} and ϕ_{g2}.

Supplementary Information 2: Basic magnetic properties of the FeGe sample

Figure S2(a-d) shows Fresnel images obtained at 230 K in the presence of different external magnetic fields of (a) 0 mT, (b) 145 mT, (c) 340 mT and (d) 500 mT. At 0 mT, the magnetic structure consists of stripe domains (helical phase) with a periodicity of 75 nm. At 145 mT, a perfectly ordered skyrmion lattice with a hexagonal structure is observed. Increasing the field, the skyrmion lattice shows more and more defects and a highly disordered phase is obtained at 340 mT. At 500 mT, a monodomain (saturated) state is reached and the remaining variations of contrast can be due to diffraction contrast, deformations of the lamella or surface inhomogeneities for instance. Figure S2(e) is a schematic diagram of the magnetic phases as a function of applied magnetic field and temperature based on a previous TEM study [3]. The Curie temperature is approximately 280 K. The skyrmion lattice pocket (shown in red) occurs slightly below the T_c and in the presence of external fields in the range of 100-300 mT. The exact shape of the phase diagram depends on the thickness of the TEM lamella [3].

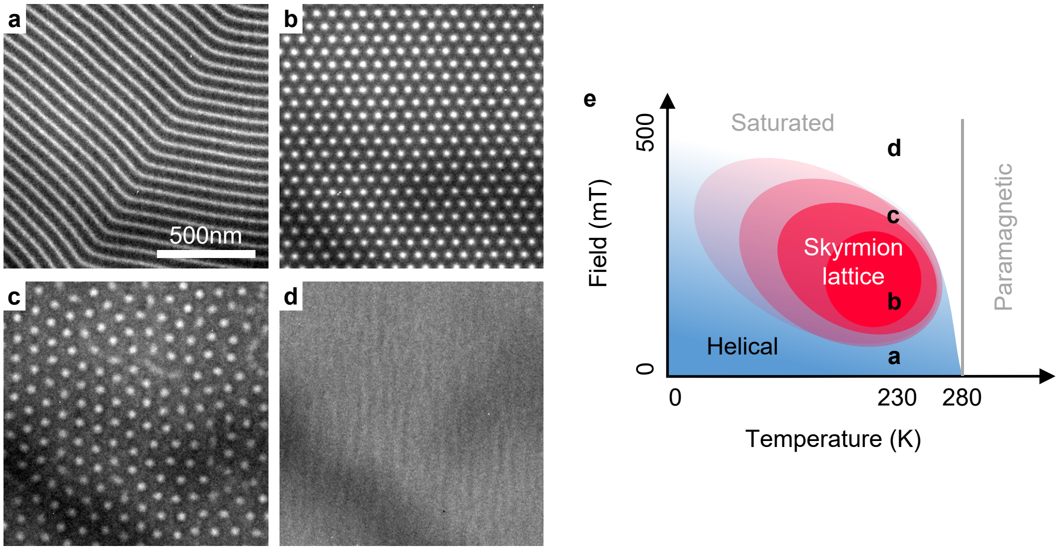

Figure S2: **Magnetic textures in FeGe.** (a-d) Fresnel images of the FeGe sample obtained at 230 K with a defocus of 800 µm and in the presence of external magnetic fields of (a) 0 mT, (b) 145 mT, (c) 340 mT and (d) 500 mT. (e) Schematic diagram of the magnetic phases as a function of temperature and external magnetic field.

Supplementary Information 3: Mapping of the local orientation parameter in real space

Figure S3(a-d) shows Fresnel images of a skyrmion lattice obtained at 145 mT, 291 mT, 326 mT and 340 mT, which are also shown in Fig. 4 of the main article. The local orientational order parameter is defined [4]

$$\Psi_6(r_i) = \frac{1}{N_i}\sum_{j=1}^{N_i} \exp(i6\theta_{ij}) \, , \tag{S9}$$

where r_i is the position of a skyrmion, N_i is the number of nearest neighbors, θ_{ij} is the angle between the line connecting the central skyrmion i and its neighbors j with respect to an arbitrary axis. In order to map Ψ_6, the Fresnel images were first filtered by applying an array of apertures to the Bragg spots in Fourier space. A sub-pixel detection of the intensity maxima was then carried out in real space and a script was used to find the nearest neighbors of each skyrmion within a chosen radius. The angles θ_{ij} were calculated from the coordinates of the skyrmions using $atan2$ functions. The complex number Ψ_6 was then calculated for each skyrmion and the values of $\Theta = \arg(\Psi_6)/6$ and $|\Psi_6|$ are mapped in Fig. S3(e-l), showing respectively the local orientation and the degree of *hexagonality.* The standard deviations δ_Θ and mean values $< |\Psi_6| >$ are indicated on the maps.

At low fields (145 mT to 326 mT), the maps obtained with this method are visually similar to the maps obtained using GPA (see Fig. 4 of the main article) and the values of standard deviation and mean are also close. At large field (340 mT), the disorder is strong and it is more difficult to compare the images visually. Information is also missing in some regions because some skyrmions show a blurry contrast, making it difficult to identify their position. Figure S3(m-t) shows the difference between the maps obtained using peak-finding and GPA. The images at low fields (145 mT to 291 mT) show essentially some random noise. At 326 mT, some differences can be seen along the grain boundary related to the presence of dislocations. These differences are amplified at 340 mT across the whole image due to the large density of defects. It might indicate that the two methods lead to different values when the lattice shows large distortions but it could also be due to the fact that in GPA the signal varies from pixel to pixel whereas here the values are constant over the interskyrmion distance.

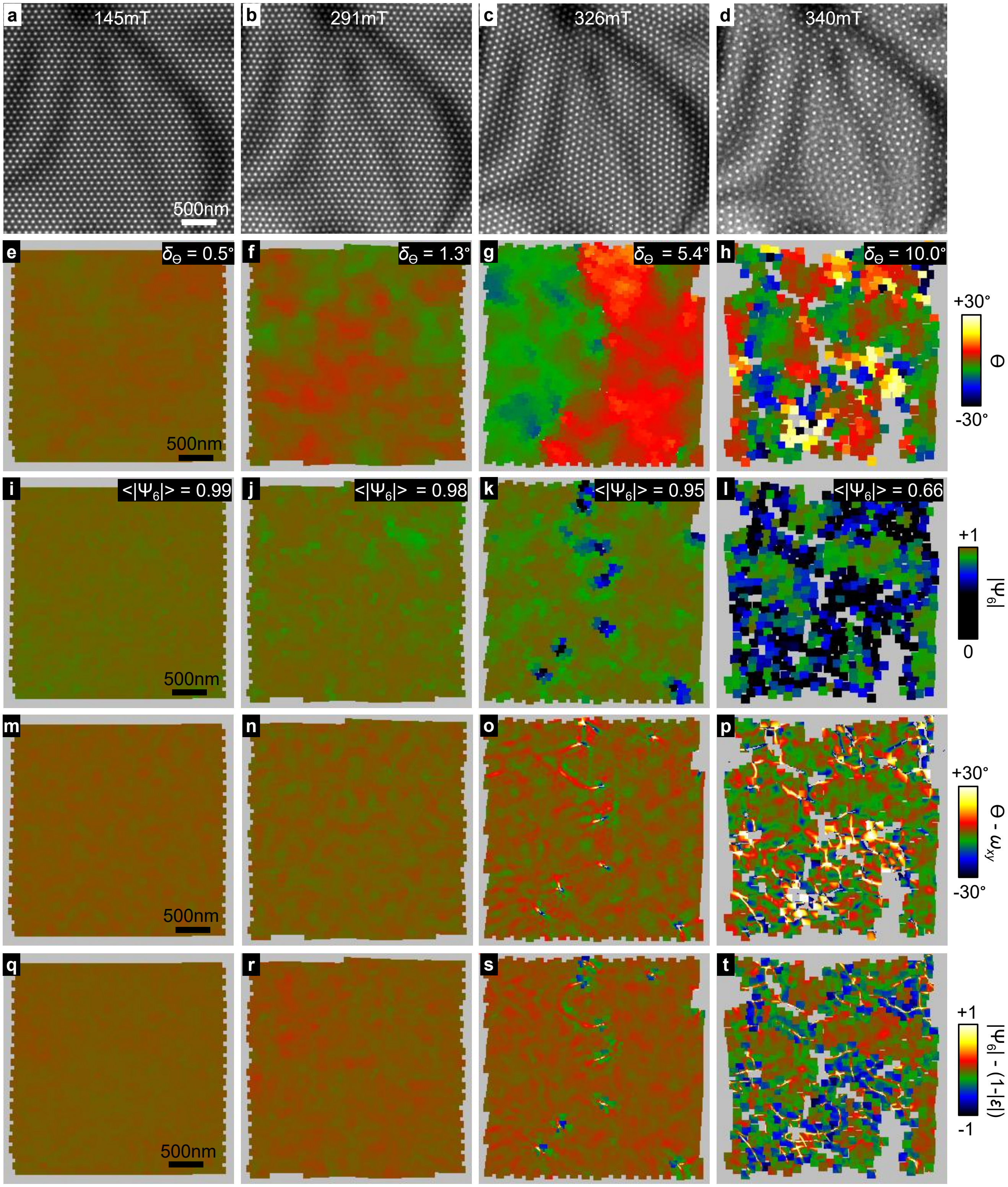

Figure S3: **Local orientational order mapping in applied magnetic field series**. (a-d) Fresnel images obtained at 230 K in the presence of external magnetic fields of 145 mT, 291 mT, 326 mT and 340 mT. (e-h) $\Theta = \arg(\Psi_6)/6$ orientation maps and (i-l) $|\Psi_6|$ *hexagonality* maps obtained in real space using a peak-finding method. The standard deviation δ_Θ and the mean value $< |\Psi_6| >$ are indicated on each map. (m-t) Difference between the maps obtained using peak-finding and GPA.

Supplementary Information 4: Influence of geometric distortions on deformation maps

Geometric distortions related to projector lenses are a possible source of artifact when measuring deformations in TEM images [5]. One way to study these distortions is to measure deformation fields across an image of a uniform crystal. The mean dilatation Δ_{xy} and the rigid-body ω_{xy} rotation maps can reveal changes in magnification and angle across the field of view [5]. Figure S4(a) shows an image of a perfect skyrmion lattice without defects and with similar optical settings (magnification, defocus) as the other images of the article. Figure S4(b) is the corresponding Fourier transform which shows sharp peaks and Fig. S4(c,d) shows the Δ_{xy} and ω_{xy} maps calculated using GPA. The two images are relatively flat and the variations observed across the images, with standard deviations of 0.6% and 0.5° respectively, are essentially due to a high frequency random noise. Figure S4(e,f) shows profiles extracted from the maps along the dashed lines. Δ_{xy} and ω_{xy} vary by approximately $\pm 1\%$ and $\pm 1°$ across the field of view, which is small compared to deformations and rotations measured locally around dislocations and grain boundaries. Therefore, we considered that the influence of geometric distortions is negligible in this study.

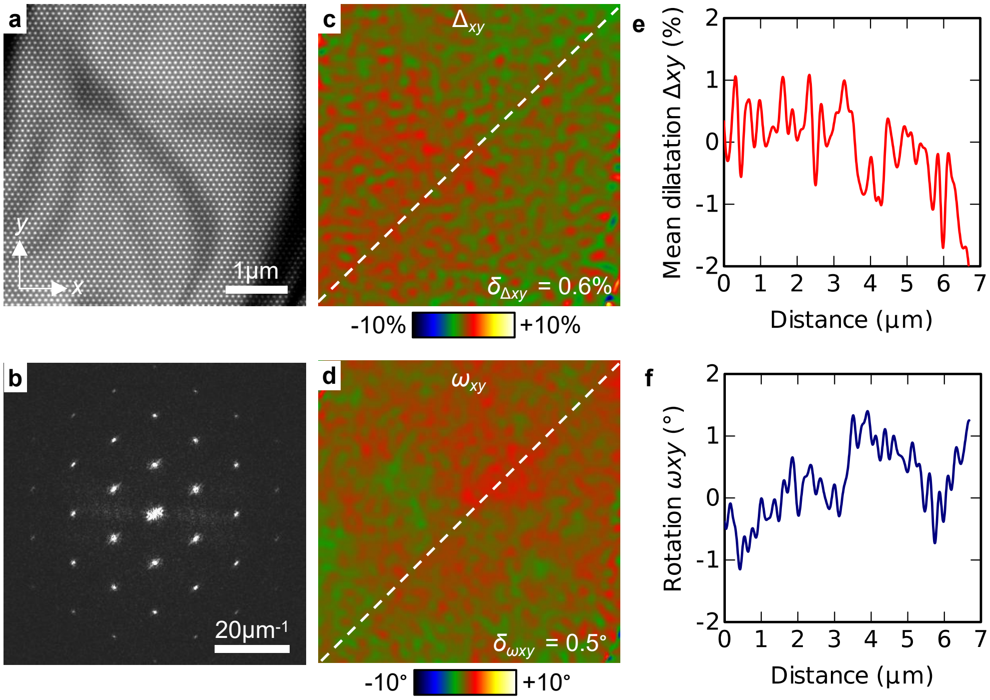

Figure S4: **Investigation of geometric distortions.** (a) Fresnel image of a perfect skyrmion lattice recorded at 230 K with a defocus of 800 μm and in the presence of an external magnetic field of 146 mT. (b) Fourier transform of (a). (c,d) Mean dilatation Δ_{xy} and rigid-body rotation ω_{xy} maps calculated using GPA. (e,f) Profiles extracted from (c,d) along the dashed lines.

Supplementary Information 5: Influence of defocus and sample thickness on deformation maps

Figure S5(a) shows the magnetization distribution (m_x, m_y, m_z) of a skyrmion lattice with a 5-7 dislocation in the middle, which was calculated using micromagnetic simulations, as described in Supplementary Information 6. The calibration of the images was adjusted so that the period of the lattice matches with the period measured experimentally in Fig. S1(a). The skyrmion size, defined as the distance between the peak and the dip in the m_y profile, is 38 nm. Figure S5(b) shows a ε_{xx} deformation map calculated from the m_z image using GPA, which is used as a reference for comparison with maps calculated from Fresnel images.

The magnetic contribution to the phase φ_{mag} was calculated in (q_x, q_y) Fourier space following

$$\varphi_{\mathrm{mag}}(x,y) = -\frac{i\pi\mu_0 M_S t}{\Phi_0}\left(\frac{\widetilde{m}_x q_y - \widetilde{m}_y q_x}{q_x^2+q_y^2}\right) , \tag{S10}$$

where μ_0 is the permeability of free space, M_S is the saturation magnetization of the sample (we used $M_S = 130$ kA.m^{-1} [6]), t is the thickness, $\Phi_0 = \frac{\pi\hbar}{e}$ is the magnetic flux quantum and $(\widetilde{m}_x, \widetilde{m}_y)$ are the Fourier transforms of the in-plane magnetization components (m_x, m_y) [7].

Fresnel images were calculated from the magnetic phase image by setting up a wave function of the form $\Psi\left(x,y\right) = e^{i\varphi_{\mathrm{mag}}(x,y)}$, which was modified in Fourier space according to

$$\Psi_{\mathrm{LTEM}}\left(x,y\right) = \mathcal{F}_2^{-1}\left\{\mathcal{F}_2\left\{e^{i\varphi_{\mathrm{mag}}(x,y)}\right\}\cdot e^{-i\chi(q_x,q_y)}\right\} , \tag{S11}$$

where $\mathcal{F}_2\left\{...\right\}$ and $\mathcal{F}_2^{-1}\left\{...\right\}$ denote a two-dimensional Fourier transform and its inverse. $\chi\left(q_x,q_y\right)$ is an aberration function, which takes the form [8]

$$\chi\left(q_x,q_y\right) = \pi\lambda\Delta f\left(q_x^2+q_y^2\right) \tag{S12}$$

and contains only the term of defocus Δf in this work. The spherical aberration of the Lorentz lens and the divergence of the electron source (damping envelope) were not taken into account. The intensity of the final Fresnel images is given by

$$I_{\mathrm{LTEM}}\left(x,y\right) = \Psi_{\mathrm{LTEM}}\left(x,y\right)\cdot\Psi^*_{\mathrm{LTEM}}\left(x,y\right) . \tag{S13}$$

To evaluate the influence of the defocus and the sample thickness, Fig. S5(c) shows Fresnel images calculated for different thicknesses of $t = 50$ nm, 150 nm and 300 nm and for different defoci of $\Delta f = \pm 0.5$ mm, ± 1 mm, ± 1.5 mm, ± 2 mm and ± 3 mm. The corresponding ε_{xx} deformation maps reconstructed using GPA are displayed next to the images. Maps that show significant deviations from the reference deformation field (in Fig. S5(b)) are indicated with a "!" symbol. Maps obtained with a defocus smaller than or equal to ± 1.5 mm are identical and in agreement with the reference field for the three different thicknesses. On the other hand, deviations can be observed when $\Delta f = -2$ mm and $\Delta f = \pm 3$ mm. For such large defoci, the reconstructed deformation field varies also with the thickness.

To determine whether thickness gradients can introduce artifacts, Fig. S6(a) shows Fresnel images of a perfect skyrmion lattice calculated with a linear thickness gradient (from $t = 50$ nm to 300 nm) and again different defoci. The corresponding ε_{xx} deformation maps are displayed next to the images and profiles extracted from the maps are shown in Fig. S6(b). For small defoci ($\leq \pm 2$ mm), no significant variations are observed across the maps. Significant variations of several percent are observed for defoci of $\Delta f = -2.5$ mm

and +3 mm, which is related to an inversion of the Fresnel contrast.

In summary, simulations show that, for defoci smaller than or equal to ±1.5 mm, deformations calculated from Fresnel images are in agreement with deformations calculated from the magnetization distribution. The sample thickness and thickness gradients have no significant influence in this range.

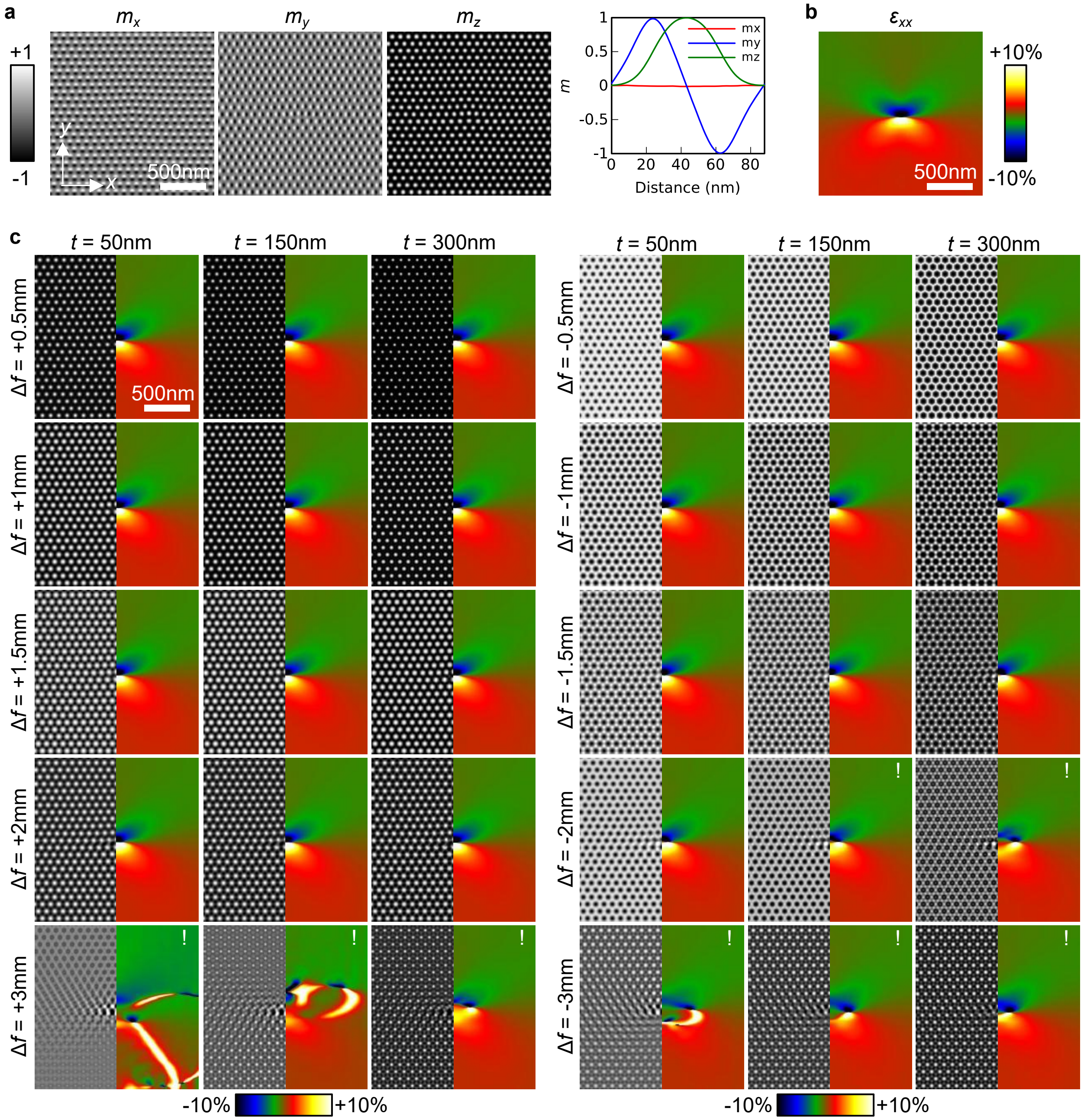

Figure S5: **Simulated defocus series for different sample thicknesses.** (a) Magnetization distribution (m_x, m_y, m_z) of a skyrmion lattice with a dislocation at the center obtained using micromagnetic simulation. Profiles were extracted from the images across a single skyrmion along the horizontal direction. (b) ε_{xx} deformation field reconstructed from the m_z image using GPA. (c) Corresponding series of Fresnel images calculated from the magnetization distribution. Three different thicknesses of $t = 50$ nm, 150 nm and 300 nm were simulated with different defoci of $\Delta f = \pm 0.5$ mm, ± 1 mm, ± 1.5 mm, ± 2 mm and ± 3 mm as indicated in the figure. Corresponding ε_{xx} deformation maps reconstructed using GPA are displayed next to the images. Only one half of the images is shown since the ε_{xx} deformation field is symmetric with respect to the central vertical axis. Images where the deformation field deviates significantly from the reference field in (b) are indicated by a “!” symbol.

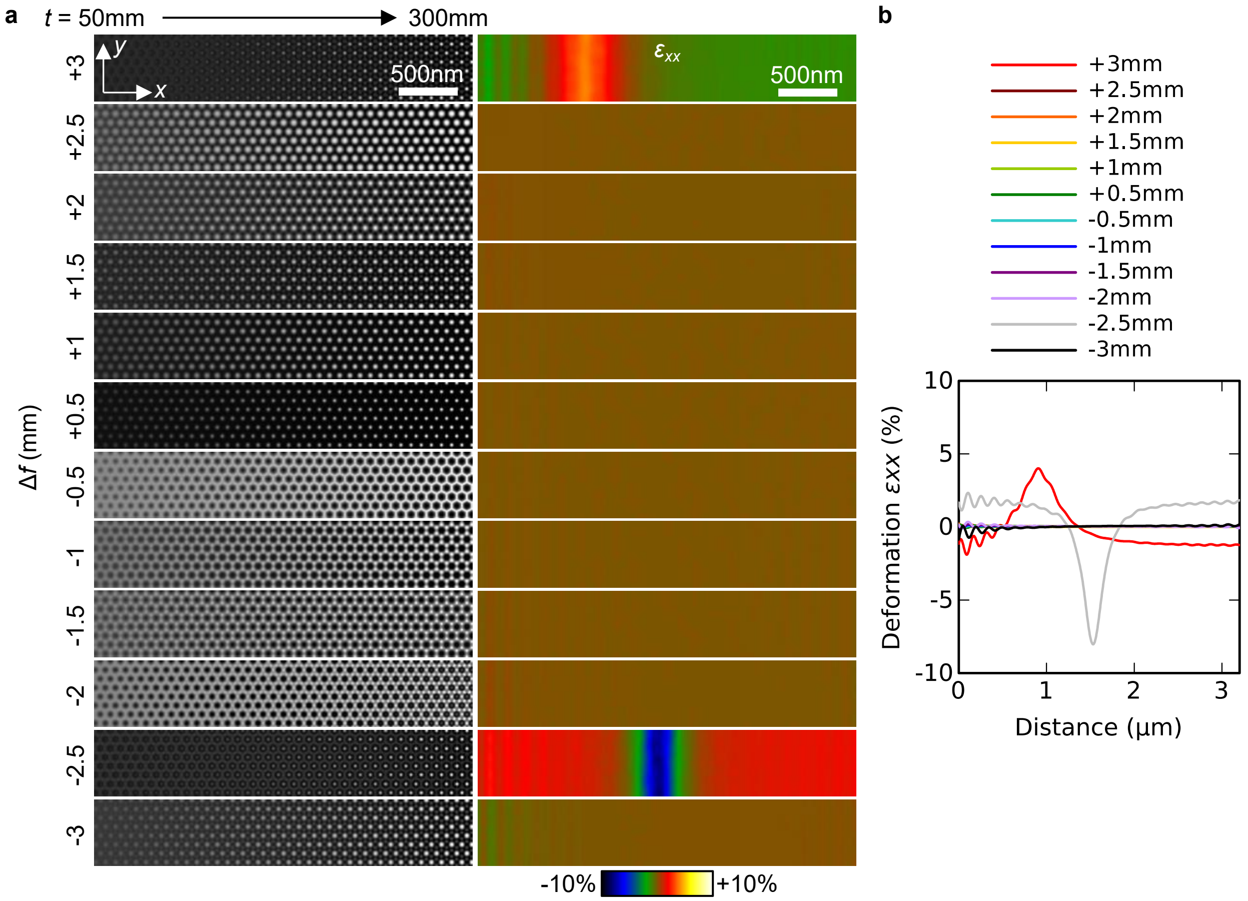

Figure S6: **Simulated defocus series with a thickness gradient.** (a) Series of Fresnel images calculated from a micromagnetic model of a perfect skyrmion lattice and corresponding ε_{xx} deformation field reconstructed using GPA. Images were calculated with a horizontal thickness gradient of 50 nm to 300 nm and with different defoci indicated in the figure. (b) Deformation profiles extracted from the maps along the horizontal direction.

Supplementary Information 6: Micromagnetic simulation of a skyrmion lattice including a dislocation

To obtain the magnetization distribution of a skyrmion lattice with a 5-7 dislocation at the center, we performed micromagnetic simulations with mumax code [9]. For exact values of material parameters such as exchange stiffness, $\mathcal{A}$, bulk DMI, $\mathcal{D}$, and saturation magnetization, M_{s} see corresponding mumax script provided below. In this simulation, we use a two-dimensional grid composed of 4096×4096 cuboids. The lateral size of the square shape domain is $L_x \times L_y = 5.44\,\mu\mathrm{m} \times 5.44\,\mu\mathrm{m}$ and thickness of the plate is $L_z = 1$ nm. In units of equilibrium period of helical modulations, $L_{\mathrm{D}} = 4\pi\mathcal{A}/\mathcal{D}$, the size of the simulated domain equals $L_x = L_y = 77.7L_{\mathrm{D}}$. The initial configuration was set such that the first half of the domain is filled by the skyrmion lattice with a period P_1, and the second half contains a skyrmion lattice with a period P_2. The period $P_1 = 90.66$ nm is set to be close to the equilibrium period of the skyrmion lattice. The period $P_2 = 92.52$ nm is selected such that there is exactly one missing plane between two skyrmion lattices. After the energy minimization at external magnetic field $B_{\mathrm{ext}} = 0.55B_{\mathrm{D}}$, where critical field $B_{\mathrm{D}} = \mathcal{D}^2/(2\mathcal{A}M_{\mathrm{s}})$, we obtained the magnetic state depicted in Fig. S5(a). Note that we use open boundary conditions in all directions, but in the figure, we show only the central part of the simulated domain, which corresponds to approximately one-quarter of the whole domain. Note also that the parameters used here are optimized for a temperature of 95 K, which leads to a small discrepancy between the skyrmion lattice period of the simulated image and the period observed experimentally at a temperature of 230 K. For this reason, the calibration of the images in Fig. S5(a) was adjusted so that the period of the skyrmion lattice matches with the experimental images.

```
Msat = 384e3      // [ A/m ]
Aex = 4.75e-12    // [ J/m ]
Dbulk = 0.853e-3 // [J/m^2]
SetPBC(0, 0, 0)
Nx := 4096
Ny := 4096
Nz := 1
SetGridsize(Nx, Ny, Nz)
SnapshotFormat="png"
Lx := 5440e-9     // [m]
Ly := 5440e-9     // [m]
Lz :=    1e-9     // [m]
SetCellsize(Lx/Nx, Ly/Ny, Lz/Nz)
P1 := Lx/60.0     // period of the lattice on the left
P2 := Lx/58.8     // period of the lattice on the right
EnableDemag = false
B := 0.11         // [T]
B_ext = vector(0, 0,B)

//Setting initial state
SkLattice := newVectorMask(nx, ny, nz)
Q11 := vector(2*pi/P1, 0, 0)
Q21 := vector(2*pi/P1*cos(pi/3), 2*pi/P1*sin(pi/3),0)
Q31 := vector(2*pi/P1*cos(pi/3),-2*pi/P1*sin(pi/3),0)
Q12 := vector(2*pi/P2, 0, 0)
Q22 := vector(2*pi/P2*cos(pi/3), 2*pi/P2*sin(pi/3),0)
Q32 := vector(2*pi/P2*cos(pi/3),-2*pi/P2*sin(pi/3),0)
```

```
F1 := atan2(Q11.y(),Q11.x())-pi/2
F2 := atan2(Q21.y(),Q21.x())-pi/2
F3 := atan2(Q31.y(),Q31.x())-pi/2
for j := 0; j < Ny; j++{
        for i := 0; i < Nx; i++{
                r := index2coord(i, j, 0)
                x := r.X()
                y := r.Y()
                T1 := 0.0
                T2 := 0.0
                T3 := 0.0
                if x < P1/2{
                        T1 = Q11.dot(r)+pi
                        T2 = Q21.dot(r)+pi
                        T3 = Q31.dot(r)+pi
                }else{
                        T1 = Q12.dot(r)+pi
                        T2 = Q22.dot(r)+0
                        T3 = Q32.dot(r)+0
                }
                nn1 := vector(cos(F1)*sin(T1),sin(F1)*sin(T1),cos(T1))
                nn2 := vector(cos(F2)*sin(T2),sin(F2)*sin(T2),cos(T2))
                nn3 := vector(cos(F3)*sin(T3),sin(F3)*sin(T3),cos(T3)+0.75)
                nn := nn1.add(nn2.add(nn3))
                nnlen := nn.len()
                nn.div(nnlen)
                SkLattice.setVector(i, j, 0, nn)
        }
}
m.SetArray(SkLattice)
// m.LoadFile("ini.ovf")
Snapshot(m.comp(0)); Snapshot(m.comp(1)); Snapshot(m.comp(2));
SaveAs(m, "ini.ovf")

MinimizerStop=1e-5
minimize()
save(m)
Snapshot(m.comp(0)); Snapshot(m.comp(1)); Snapshot(m.comp(2));
```

References

[1] Hÿtch, M. J. & Gandais, M. Quantitative criteria for the detection and characterization of nanocrystals from high-resolution electron microscopy images. *Philos. Mag. A* **72**, 619–634 (1995). URL https://doi.org/10.1080/01418619508243789.

[2] Hÿtch, M. J., Snoeck, E. & Kilaas, R. Quantitative measurement of displacement and strain fields from HREM micrographs. *Ultramicroscopy* **74**, 131–146 (1998). URL https://doi.org/10.1016/S0304-3991(98)00035-7.

[3] Yu, X. Z. *et al.* Near room-temperature formation of a skyrmion crystal in thin-films of the helimagnet FeGe. *Nat. Mater.* **10**, 106–109 (2010). URL https://doi.org/10.1038/NMAT2916.

[4] Nelson, D. R. & Halperin, B. I. Dislocation-mediated melting in two dimensions. *Phys. Rev. B* **19**, 2457–2484 (1979). URL https://doi.org/10.1103/PhysRevB.19.2457.

[5] Hüe, F. *et al.* Calibration of projector lens distortions. *Microscopy* **54**, 181–190 (2005). URL https://doi.org/10.1093/jmicro/dfi042.

[6] Song, D. *et al.* Quantification of magnetic surface and edge states in an FeGe nanostripe by off-axis electron holography. *Phys. Rev. Lett.* **120**, 167204 (2018). URL https://doi.org/10.1103/PhysRevLett.120.167204.

[7] Beleggia, M. *et al.* Quantitative study of magnetic field distribution by electron holography and micromagnetic simulations. *Appl. Phys. Lett.* **83**, 1435–1437 (2003). URL https://doi.org/10.1063/1.1603355.

[8] Chapman, J. N. The investigation of magnetic domain structures in thin foils by electron microscopy. *J. Phys. D: Appl. Phys.* **17**, 623–647 (1984). URL https://doi.org/10.1088/0022-3727/17/4/003.

[9] Vansteenkiste, A. *et al.* The design and verification of MuMax3. *AIP Adv.* **4** (2014). URL https://doi.org/10.1063/1.4899186.
